# Aptamer Based on Silver Nanoparticle-Modified Flexible Carbon Ink Printed Electrode for the Electrochemical Detection of Chikungunya Virus

**DOI:** 10.3390/bios14070344

**Published:** 2024-07-16

**Authors:** Pradakshina Sharma, Mohd. Rahil Hasan, Ubaid Mushtaq Naikoo, Shaheen Khatoon, Roberto Pilloton, Jagriti Narang

**Affiliations:** 1 Department of Biotechnology, School of Chemical and Life Sciences, Jamia Hamdard, Hamdard Nagar, New Delhi 110062, India; pradakshinasharma@gmail.com (P.S.); rahilhasan789@gmail.com (M.R.H.); ubaidnaik11@gmail.com (U.M.N.); kshaheen917@gmail.com (S.K.); 2 Institute of Crystallography, National Research Council, 00143 Rome, Italy

**Keywords:** flexible substrate, electrochemical, chikungunya virus, aptasensor

## Abstract

Medical devices have progressed from their initial bulky forms to smart devices. However, their rigidity hampers their seamless integration into everyday life. The fields of stretchable, textile, and flexible electronics are emerging research areas with the potential to drive significant technological progress. This research presents a laboratory-based technique to produce highly sensitive and flexible biosensors for detecting the chikungunya virus. These biosensors are based on 0D nanomaterials and demonstrate significant advancements in voltammetry. The electrochemical platform was created utilizing the stencil printing (StPE) technique. Adapting the biosensor setup involved the selection of aptamer as the biorecognition element bound with silver nanoparticles (AgNPs). This biosensor was employed in the voltammetric identification of the Chikungunya virus antigen (CHIKV-Ag) within a solution containing 0.5 mM potassium ferro/ferri cyanide, a redox pair. The biosensor was employed to evaluate CHIKV-Ag within a human serum sample. It demonstrated a linear detection span ranging from 0.1 ng/mL to 1 μg/mL, with a detection limit of 0.1 ng/mL for CHIKV-Ag. The proposed approach, due to its flexibility in production and the electrocatalytic attributes displayed by the zero-dimensional nanostructure, presents innovative opportunities for cost-effective and tailored aptamer-based bioelectronics, thereby broadening the scope of this domain.

## 1. Introduction

Chikungunya virus (CHIKV) is an alphavirus transmitted by mosquitoes, which has resurged and caused notable outbreaks of disease in areas including the Indian Ocean islands, Southeast Asia, and the Americas since 2004 [1]. Individuals affected by CHIKV experience incapacitating musculoskeletal ailments, including fever, rash, polyarthralgia, and myalgia [2]. Typically, this condition shows symptoms that are self-limiting and not life-threatening. However, some patients may experience uncommon or severe clinical signs, leading to the development of chronic rheumatic disorders [3]. According to the World Health Organization (WHO), no available vaccines or antiviral treatments have been proven effective against CHIKV. The observable signs of this virus might be erroneously attributed to other infectious agents, such as the dengue virus, thereby complicating the diagnosis process [4]. Over the past few decades, various established methods for disease diagnosis have been documented, including immunological approaches like enzyme-linked immunosorbent assays (ELISA) and viral-RNA identified utilizing polymerase chain reaction (PCR) methods. However, these approaches lack sensitivity or specificity, necessitate expert interpretation, consume substantial time, and use expensive and intricate equipment. Given these constraints, these techniques are unsuitable for regions with limited resources and personnel, underscoring the need for further research to propel the evolution of biosensors for Chikungunya detection [5]. In recent times, there has been a notable increase in the market demand for intelligent analytical biosensors, particularly in settings such as Point-of-Care (PoC) or Point-of-Need (PoN) [6,7,8]. A significant benefit of electrochemical devices is their capacity to produce compact and portable analytical tools, facilitating on-site analysis and diagnostics [9,10,11]. Under these conditions, the biomedical analysis sector is transitioning towards producing devices using cost-effective laboratory-scale or compact instrumentation, ensuring they meet the necessary performance standards.

Continuous advancements in technology and methods have been incorporated to improve the capabilities and usability of the devices that have been developed [12]. In addition to serving as a framework for building sensors and biosensors, one-time-use electrodes have frequently been utilized in complex sample analysis [13]. The advancement of disposable devices relies on their economic viability and the ability to be mass-produced while preserving their fundamental characteristics [14]. In this case, serigraph printing or stencil printing methods are distinguished for their plainness and framework, which enable a wide variety of sensor designs [15,16,17].

Aptasensors, a type of electrochemical biosensor, stand out for their ability to detect unique characteristics not typically targeted by other biosensors. Aptamers are small molecules that bind to proteins, nucleic acids, and other compounds. Aptamers possess a distinctive oligonucleotide sequence, displaying significant binding strength and exceptional specificity towards a range of substances, such as proteins, peptides, small molecules, and even cells [18,19,20]. The advantages of aptamers compared to natural receptors like antibodies and enzymes as biometric probes include their chemical durability, smaller size, and cost-efficiency [21]. Consequently, aptamers have emerged as promising foundations for advancing sensors due to their simple generation process and remarkable specificity and affinity towards targets [22,23].

Moreover, the performance of electrochemical biosensors is notably affected by the sensitive materials applied to the working electrode’s surface. Therefore, various materials can be utilized to develop a functional electrode surface that enhances the binding of CHIKV antigen. This enhancement can improve the sensitivity and selectivity of the sensors, enabling more efficient detection of CHIKV.

Metal nanoparticles are an excellent choice due to their non-toxic nature and ease of production. Silver nanoparticles (AgNPs) offer distinct benefits for electrochemical bio-sensing due to their exceptional characteristics, including substantial surface area, electrical conductivity, and compatibility with biological systems, resulting in numerous advantages [24,25].

Additionally, the electrodes can be fabricated using various chemically stable surfaces [26], such as paper, textiles, polymer materials, glass, and ceramic substrates [27]. The choice of substrate material is typically determined by the specific application of the sensor, with consideration given to its disposability after use [28]. Flexible plastic and cellulosic substrates have demonstrated their benefits in advancing electroanalytical devices because of their remarkable adaptability, presenting opportunities beyond conventional screen-printing techniques [29]. Polyvinyl chloride (PVC) is a highly demanded polymer worldwide, as evidenced by its extensive production and consumption. Its large production volume ensures easy access to retail markets, and its exceptional mechanical and chemical properties, widespread availability, and hydrophobic characteristics make it suitable for sensor fabrication purposes [30,31].

As a result, a PVC-nano-based aptasensor could significantly improve signal detection with increased sensitivity and resolution, establishing it as the most dependable technique for identifying the Chikungunya virus Antigen. This approach is suitable for implementation in developing countries because of its affordability and minimal sample preparation prerequisites [32,33]. This study utilized a simple detection technique employing a three-electrode configuration. The PVC-based electrode was modified using AgNPs. Further, the immobilization of the aptamer can be executed through diverse strategies, depending on the sensing interface. These methods include covalent immobilization via NH_2_ and carboxyl or thiol linkages [33].

Furthermore, cross-linking can be achieved using glutaraldehyde [34]. Typically, covalent bonds are favored for attaching biorecognition elements to metal substrates like FTO or gold-based electrodes, primarily when these electrodes are intended for reuse after regeneration. However, we opted for adsorption [35] for immobilizing aptamer in this study. Although this method may not offer the most vital type of immobilization, it proves highly effective in developing disposable biosensors. Numerous studies have documented the use of adsorption for immobilizing aptamer [36,37].

Moreover, a potentiostat was employed to conduct all electrochemical measurements. Characterization of nanoparticles was performed using Transmission Electron Microscopy (TEM), Surface electron microscopy (SEM), Fourier transform infrared spectroscopy (FTIR), Atomic force microscopy (AFM), and UV-Vis spectroscopy. Cyclic voltammetry (CV), linear sweep voltammetry (LSV), and electrochemical impedance spectroscopy (EIS) utilizing Ferro/Ferricyanide as a redox couple material are particularly advantageous for the identification of CHIKV-Antigen (CHIKV-Ag). This study aimed to provide a potential early CHIKV-Antigen (Ag) detection framework.

Due to the similarity of clinical symptoms between the current disease under investigation and other conditions, an additional assessment was conducted to evaluate its cross-reactivity. It was tested utilizing a non-related antigen derived from the dengue virus. The aptasensor was additionally assessed using human serum samples, demonstrating satisfactory performance. The novel detection method proposed is accurate and time-efficient and holds the potential for integration into state-of-the-art technology. This technique could identify CHIKV-infected individuals in hospital and laboratory environments.

## 2. Methods and Materials

### 2.1. Reagents and Materials

Silver nitrate and sodium borohydride were procured from GLR invention to synthesize silver nanoparticles. Potassium Ferro/ferri cyanide was acquired from LOBA CHEMIE (Maharashtra, India). The Chikungunya antigen was procured from Prospec (Rehovot, Israel) and dissolved in 10× phosphate-buffered saline (PBS) with a pH of 7.4 to make up the 1.95 mg/mL of the antigen. Further, various dilutions of CHIKV-Ag stock solution were prepared to range as 0.1 ng/mL, 1 ng/mL, 10 ng/mL, 100 ng/mL, and 1 µg/mL in PBS at pH 7.4. Praveen Scientific (Delhi, India) supplied the polyvinyl chloride sheet. The CHIKV-aptamer was sourced from IDT Pvt Ltd. (Coralville, IA, USA). Below is the sequence.

ATCCGTCACACCTGCTCTTATCAGAAGGGGGCAGAGGAGATGTAGTGCGGGTTTTGGTGTTGGCTCCCGTAT [38].

Conductive inks such as carbon and silver ink were used and procured from SNAP Graphics Pvt. Ltd. (Bengaluru, India) to print the electrodes.

### 2.2. Instrument

A Potentiostat of Dropsens (Stat-I 400s) (Metrohm, Herisau, Switzerland) was used to conduct electrochemical measurements. Techniques including CV, LSV, and EIS were employed. UV-visible absorbance measurements were conducted using the Agilent Cary100 series spectrometer (Agilent Technologies, Santa Clara, CA, USA). The morphology of AgNPs was analyzed using Transmission Electron Microscopy (TEM) on a Talos L120C (Thermo Scientific, Waltham, MA, USA), FTIR was performed on Bruker Tenser 37 (Bruker, Billerica, MA, USA), and SEM on ZIESS EVO-18 (Zeiss, Jena, Germany).

### 2.3. Silver Nanoparticles Synthesis

A chemical-reduction procedure was employed to prepare AgNPs, which required some modifications. Gradually, 10 mL of a 1 mM AgNO_3_ solution was introduced into 30 mL of a 2 mM ice-cold freshly prepared NaBH_4_ solution with stirring constantly. The addition was performed at a rate of 1 drop per second until complete dissolution of AgNO_3_ occurred, forming a brilliant yellow solution. This bright yellow solution includes AgNPs [39].

### 2.4. PVC-Based Biosensor Fabrication and Working

**Scheme 1:**
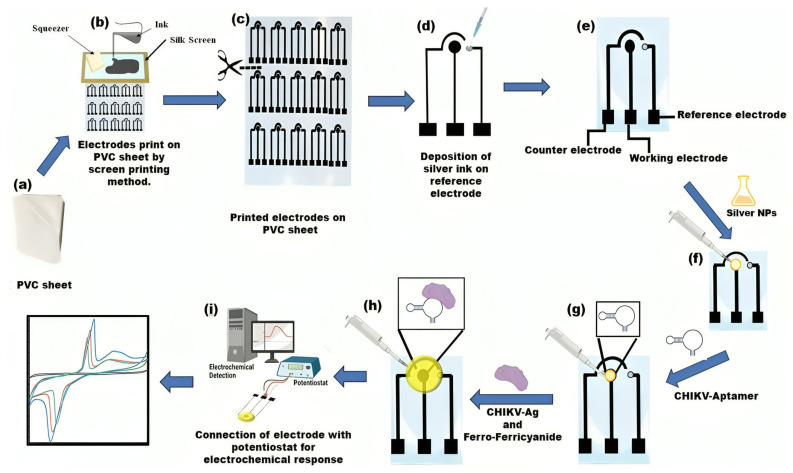
Graphical depictions illustrating the operation of electrochemical biosensors include the creation and assembly of flexible platforms. (**a**–**c**) A PVC sheet was positioned beneath the screen as a base; carbon conductive ink was dispensed and spread onto the screen, serving as a foundation for printing electrodes; (**d**) Silver conductive ink was utilized to create the reference electrodes, thereby completing the PVC-based 3-electrode system. (**e**) The PVC sheet-based setup incorporates three electrodes: the working electrode (WE), reference electrode (RE), and counter electrode (CE). (**f**) Silver nanoparticles (AgNPs) are applied to the working region of the electrode. (**g**) Aptamer immobilization on AgNPs/PVC substrate electrode. (**h**) Drop casting the CHIKV-Ag, and Ferro/ferricyanide solution. (**i**) Electrochemical analysis was conducted using a potentiostat device.

A biosensor device constructed from PVC was manufactured employing a silkscreen technique and carbon and silver conductive ink. The silkscreen, accompanied by a printed electrode stencil, functions as a template for electrode preparation. Black carbon conductive ink was used to print electrodes, and silver conductive ink was used for the reference electrode. The ink was carefully applied and squeezed onto the silk screen frame, allowing for printing the 3-electrode paper analytical device with predefined dimensions. The PVC electrochemical-based analytical device (PCE) comprises three electrodes: a reference electrode (RE), counter electrodes (CE), and working electrodes (WE). Silver conductive ink was deposited on the RE (Scheme 1). In this study, a configuration of electrodes of uniform size was established and tested. Their geometric surface area is 3 × 1.6 cm. The sensor’s workings are also illustrated in Scheme 1.

### 2.5. Electrochemical Identification of CHIKV-Ag on the Surface of the Working Electrode

On the WE of the biosensor, 30 µL of AgNPs were drop-deposited initially. Subsequently, 20 µL of aptamer (1 µM) was immobilized on a circular region of the working electrode decorated with AgNPs. Further, utilizing a micropipette, 20 µL of various array concentrations of CHIKV antigen were drop deposited on the working electrodes. CV, LSV, and EIS were conducted in 0.5 mM Ferro/Ferricyanide for analysis.

### 2.6. Other Optimization in Electrochemical Measurements

Blood (human) samples were collected from healthy volunteers and spun at 2800 revolutions per minute (rpm) for 15 min to separate the serum. These samples were transferred into vacutainer serum tubes and stored at −20 °C until they were ready for further analysis. Following this, the serum samples were subjected to spikes in the concentration of CHIKV-Antigen. The responses of the aptasensor to sera containing CHIKV antigens were compared. Subsequently, the sensor’s repeatability was assessed through multiple weekly evaluations under identical conditions to verify consistent current readings.

## 3. Results and Discussion

### 3.1. AgNPs Characterizations

**Figure 1:**
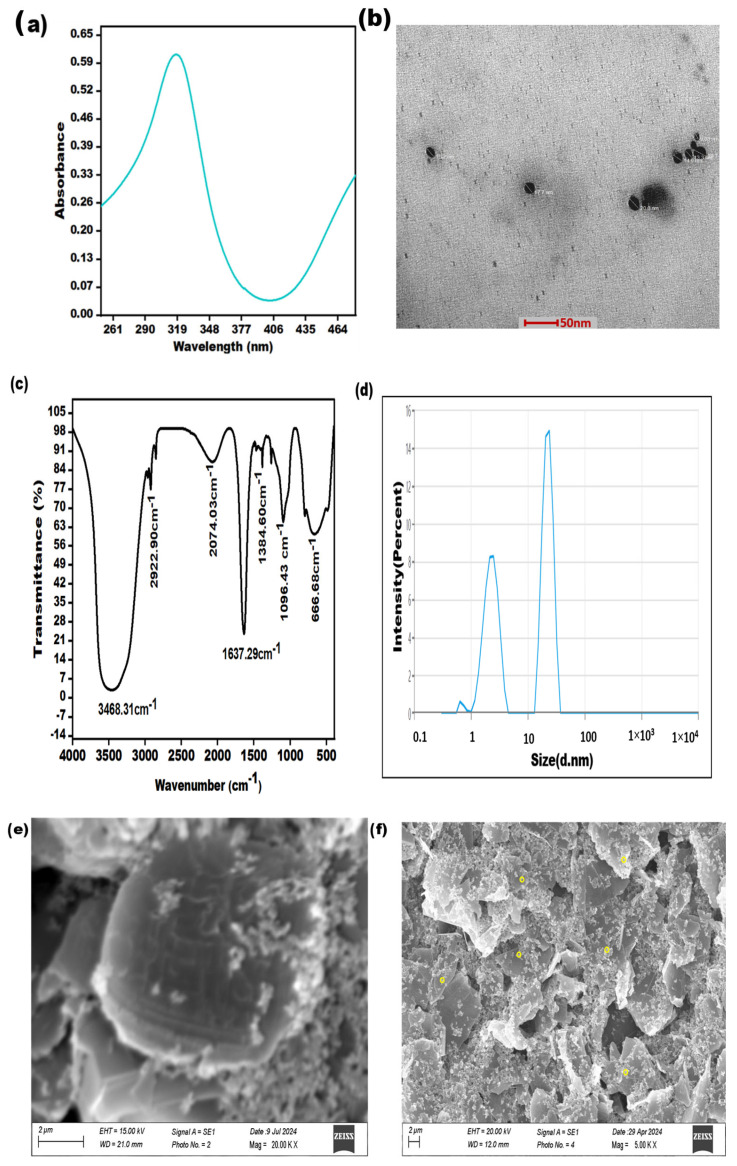
Characterization of AgNPs: (**a**) UV-visible spectroscopy (**b**); transmission electron microscopy at a 50 nm scale; (**c**) FTIR analysis; (**d**) DLS analysis; (**e**) SEM image at a scale of 02 µm of graphene carbon electrode; (**f**) SEM image at a scale of 2 µm of yellow circles denoting AgNPs, distributed on graphene carbon electrode.

The effective formation of AgNPs was validated using the subsequent characterization methods, i.e., TEM, SEM, FTIR, and UV–Vis spectroscopy. These techniques represent the fundamental and straightforward approaches to validate the generation of NPs. The distinct optical characteristics of silver nanoparticles, typically influenced by their shape and size, were examined through UV–visible spectroscopy. The synthesis of AgNPs was confirmed by observing an apparent change in the color of the silver solution, transitioning from colorless to a noticeable yellow or brown color. The UV-Vis absorption spectra display distinct peaks at approximately 317 nm, thus confirming the successful formation of silver nanoparticles, as given in Figure 1a. The morphology of AgNPs was verified by utilizing TEM, as shown in Figure 1b.

The synthesized silver nanoparticles were also subjected to FTIR spectroscopy for characterization. The spectral data were recorded in the wavelength range of 4000–500 cm^−1^. The FTIR spectrum of the synthesized silver nanoparticles exhibited peaks at 3468.31, 2922.90, 2074.03, 1384.60, 1096.43, and 666.68 cm^−1^. The peak observed at 3468.31 cm^−1^ was attributed to the stretching vibrations of the hydroxyl group. The smaller peaks at 2922.90 cm^−1^ and 2074.03 cm^−1^ were due to the stretching of the C–H bond. The sharp absorbance band at 1637.29 cm^−1^ could be assigned to N–H stretching. The presence of the peak at 1384.60 cm^−1^ indicated C–N stretching due to amino entities, while the peak at 1096.43 cm^−1^ corresponded to C–O stretching. A small peak observed at 666.68 cm^−1^ was associated with the Ag–O bond (Figure 1c). Additionally, Dynamic Light Scattering (DLS) analysis revealed that the average particle size of the silver nanoparticles fell within the range of 16–25 nanometers (Figure 1d). Furthermore, Figure 1e shows the SEM image of the unmodified electrode, and Figure 1f shows that the yellow circles denote silver nanoparticles, which distributed on a graphene carbon conductive ink-based electrode.

### 3.2. Analysis of Different Stages of Aptasensor (Aptamer/AgNPs/PCE)

**Figure 2:**
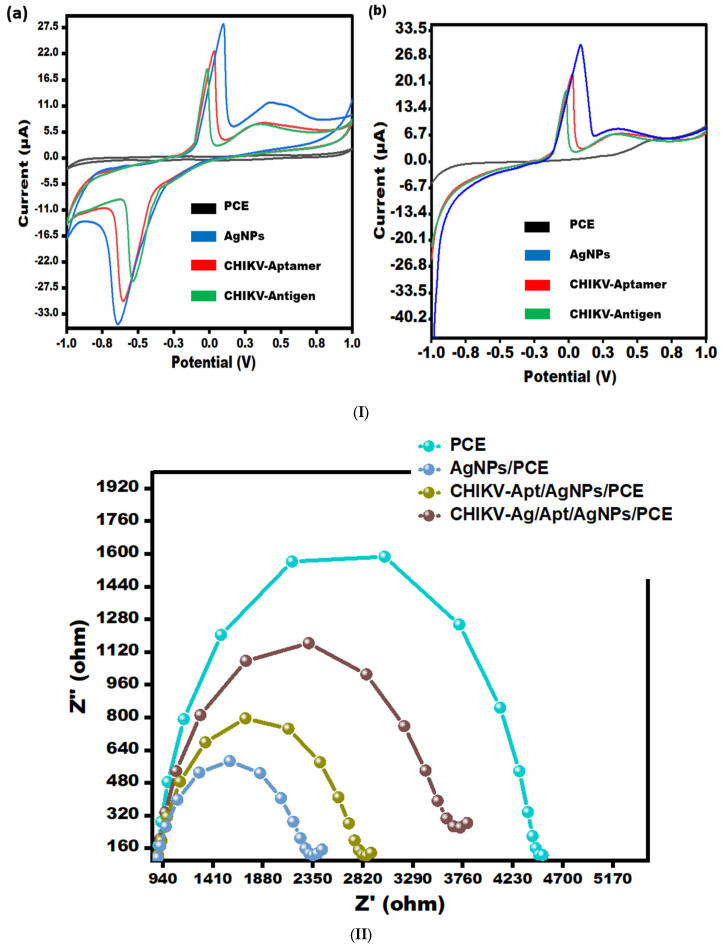
(**I**) (**a**) CV for bare-PCE, AgNPs/PCE, Aptamer/AgNPs/PCE, and CHIKV-Ag/Aptamer/AgNPs/PCE in 0.5 mM potassium ferrocyanide and ferricyanide at 50 mV/s in the potential range from −1.0 V to +1.0 V. (**b**) LSV for bare-PCE, AgNPs/PCE, Aptamer/AgNPs/PCE, and CHIKV-Ag/Aptamer/AgNPs/PCE in 0.5 mM potassium ferrocyanide and ferricyanide at 50 mV/s in the electrochemical potential range from −1.0 V to +1.0 V. (**II**) EIS spectra depicting bare-PCE, AgNPs/PCE, Aptamer/AgNPs/PCE, and CHIKV-Ag/Aptamer/AgNPs/PCE in 0.5 mM potassium ferrocyanide and ferricyanide with a frequency range from 0.1 Hz to 100 kHz.

Electrochemical analysis of developed aptasensor stages was performed via effective methods, i.e., CV/LSV (Figure 2(Ia,Ib)) and as well as EIS (Figure 2II).

#### 3.2.1. Analysis of Aptasensor Using Cyclic Voltammetry (CV) and Linear Sweep Voltammetry (LSV)

CV and LSV scans were performed to evaluate the electrocatalytic performance of the various modified sensors in a [Fe(CN)_6_]^3−/4−^ solution. These scans were conducted at a scan rate of 50 mV/s, covering a potential range from −1.0 V to 1.0 V (see Figure 2I). The peak current observed in the AgNPs/PCE curve demonstrates an apparent enhancement compared to the bare PCE curve, suggesting an improvement in the electro-conductivity of PCE due to the incorporation of silver nanoparticles. This occurrence can be ascribed to the remarkable conductive characteristics of AgNPs, enabling the effective electron transfer of [Fe(CN)_6_]^3−/4−^ species on the sensing surface. Following the immobilization of aptamer onto AgNPs, a decline in peak current was observed in both CV (Figure 2(Ia)) and LSV (Figure 2(Ib)), which can be attributed to the role of the aptamer as an insulating barrier, impeding the electron transfer process. Further, when the CHIKV antigen (0.1 ng/mL) was drop-casted, the current responses decreased significantly, suggesting that the surface of the electrode was blocked, limiting electron transit. The results obtained from CV and LSV have substantiated the observations made regarding the performance of the PCE (Figure 2I).

#### 3.2.2. Electrochemical Impedance Spectroscopy (EIS)

Electrochemical Impedance Spectroscopy (EIS) was utilized to investigate the surface properties of the modified electrode, specifically the aptamer/AgNPs/PCE configuration (as depicted in Figure 2II). The impedance spectra exhibited two discernible regions: a semicircle in the high-frequency region, indicative of electron transfer processes, and a linear section in the low-frequency region. The semicircle represents the electron transfer mechanism, with its diameter directly reflecting the charge-transfer resistance (Rct). Notably, the bare electrode exhibited a higher Rct value (4.475 kΩ) compared to the AgNPs-modified electrode (Rct = 2.315 kΩ), indicating enhanced electron transfer properties with AgNPs modification. Upon aptamer immobilization, a decrease in active sites led to an increase in electrode surface resistance, resulting in an Rct value of 2.817 kΩ. Subsequent exposure to CHIKV-Antigen caused a further increase in impedance signal as the antigen (0.1 ng/mL) bound to the aptamer, resulting in an Rct value of 3.67 kΩ. This binding event hindered electron transport, slowing down the charge transfer kinetics of the [Fe(CN)6]^3−/4−^ redox pair attached to the surface. The CV and LSV results corroborate the findings from the EIS analysis.

### 3.3. Effect of Temperature and Time on the Fabricated Sensor

**Figure 3:**
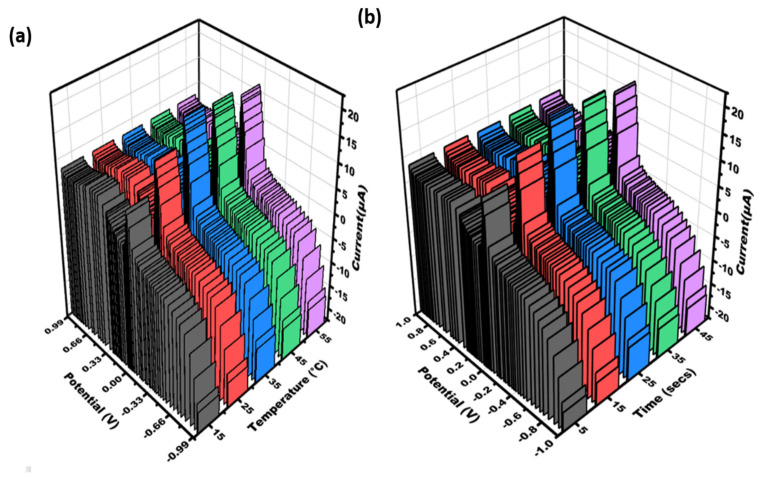
Three-dimensional visualization was generated to illustrate the cyclic voltammetry data obtained for varying concentrations of CHIKV-Aptamer/AgNPs/PCE: (**a**) temperature (15, 25, 35, 45, 55 °C); (**b**) time (5–45 s) in 0.5 mM potassium ferrocyanide and ferricyanide at 50 mV/s in the range of potential of −1 V − +1 V.

The sensor was calibrated for experimental variables to achieve the optimal response. Additionally, the impact of varying temperatures on sensor signals was investigated to attain the most effective readings. The temperature ranges between 15 and 55 degrees Celsius, and the optimum sensing signal was observed at 35 degrees Celsius (refer to Figure 3a). The sensitivity of CHIKV first increases from 5 to 25 s, and then it decreases slightly after 25 s to 45 s (see Figure 3b). Therefore, at 25 s, the modified electrode showed the maximum response.

### 3.4. Study of Different Chikungunya Virus-Antigen Concentrations on the Aptamer/AgNPs/PCE

**Figure 4:**
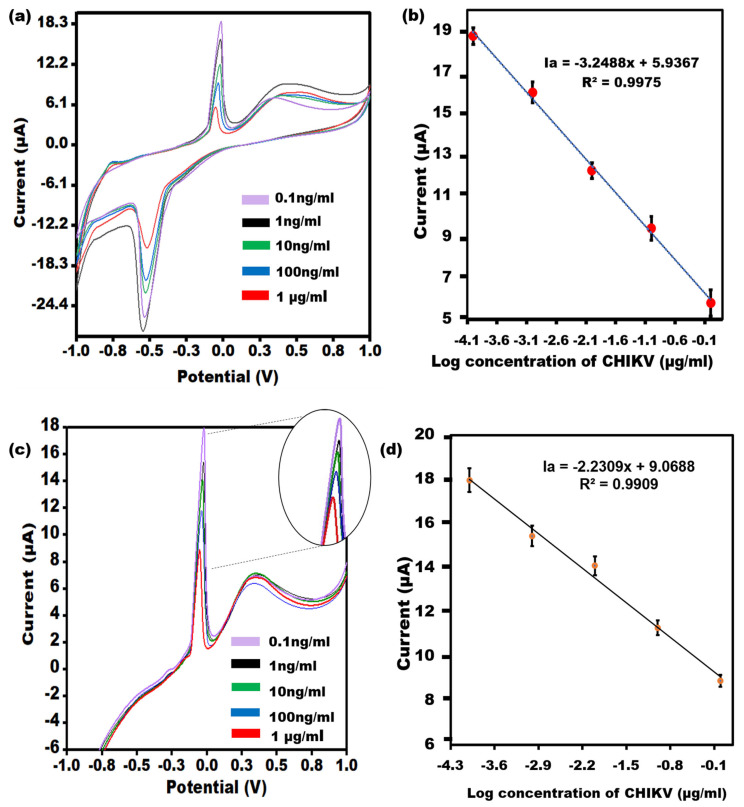
(**a**) CV of the Aptamer/AgNPs/PCE across various concentrations of CHIKV-Ag, covering linearity from 0.1 ng/mL to 1 µg/mL. (**b**) The error bars (*n* = 5) represent the CV results’ standard deviation from the aptasensor for each concentration. (**c**) LSV of the Aptamer/AgNPs/PCE across different concentrations of CHIKV-Ag, spanning a dynamic range from 0.1 ng/mL to 1 µg/mL with an enlarged view of peak current. (**d**) The error bars (*n* = 5) denote the standard deviation of the LSV results obtained from the aptasensor for each concentration.

An evaluation of the efficacy of the Aptamer/AgNPs/PCE by utilizing CV and LSV will quantify varying concentrations of CHIKV is given in Figure 4a. As the CHIKV concentration increased, the modified electrode responses became less sensitive. Additional insulating layers have been cast into the biological recognition element, which may account for the slowed electron transit. A linear relationship between the different concentrations in the peak currents is Ia = −3.2488x + 5.9367, R^2^ = 0.9975 (Figure 4b), and the concentration of CHIKV was obtained in the range of 0.1 ng/mL^−1^ μg/mL with a LOD of 0.1 ng/mL. The results of the LSV measurement (Figure 4c) confirmed the CV outcomes with the linear relationship as Ia = −2.2309x + 9.0688, R^2^ = 0.99 (Figure 4d).

### 3.5. Other Optimization Parameters of the Apta Sensor

#### 3.5.1. Recovery and Precision

To assess the analytical recovery, we utilized a technique wherein known concentrations of CHIKV-Ag (0.0005 μg/mL, 0.005 μg/mL, and 0.05 μg/mL) were added to solutions containing CHIKV-Ag/Aptamer/AgNPs/PCE at concentrations of 0.0001 μg/mL, 0.001 μg/mL, and 0.01 μg/mL, respectively. According to the findings, employing an innovative approach for CHIKV identification could lead to a successful outcome (refer to Table 1). Furthermore, the precision of the current sensor was deduced and is presented in Table 2.

#### 3.5.2. Specificity Studies, Stability, Reproducibility, and Flexibility

**Figure 5:**
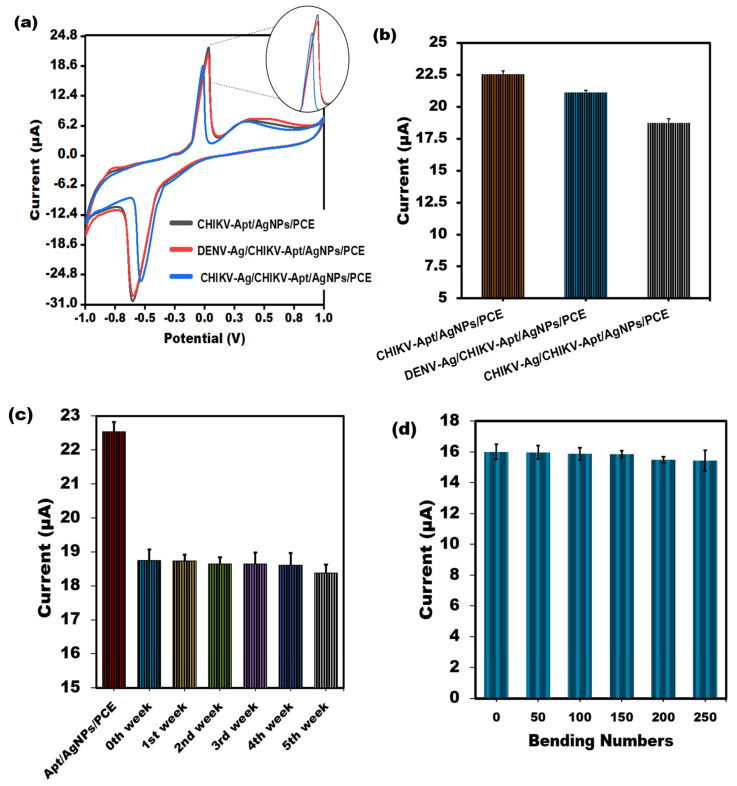
(**a**) Assessment of the CV peak current value through the binding of CHIKV-Aptamer with interferent Dengue virus Antigen using the developed PVC sheet-based sensor. (**b**) A bar graph illustrating the non-cross-reactivity of the sensor towards Dengue virus Antigen, accompanied by error bars denoting the standard deviation (*n* = 5). (**c**) The electrochemical test was used for several weeks to ascertain whether the shelf life of the built–in PVC sheet–based aptasensor would remain stable. (**d**) Effect of bending numbers on the cyclic voltammetric response of the PVC sheet-based sensor towards 1 ng/mL CHIKV-Antigen.

To evaluate the spectrum of sensor responses, PVC sheet-based aptasensors for CHIKV-Ag were tested with unrelated viral proteins, such as the Dengue virus Antigen (0.1 ng/mL). As depicted in Figure 5a,b, the sensor’s reactivity to the CHIKV-Ag (0.1 ng/mL) on the PVC sheet-based sensor is notably diminished compared to that of the non-specific protein, primarily because of the sensor’s selectivity. Strong signals for non-specific proteins showed the most significant amount of aptamer molecules attached to the surface due to the absence of binding and consequent lack of competition. Furthermore, after being stored at room temperature for several weeks, the stability of the modified sensor was assessed, and 99% of its original response was maintained. After a few weeks, 98% of its actual worth remained (Figure 5c). These results suggest the enduring stability of the sensor tailored for CHIKV. The biosensor showed good reproducibility in the CHIKV-Ag measurements, as indicated by its RSD value of below 3.0%. The exceptional flexibility of the aptasensor is crucial, particularly when employing PVC substrates to create compact lab-on-a-chip devices and cutting-edge biomedical analysis tools. Our study examined how mechanical stress from bending affects the cyclic voltammetry response of the sensor in a solution containing 1 ng/mL of CHIKV-Ag. Figure 5d demonstrates that even after bending the electrode inward 90° a total of 250 times, the cyclic voltammetric response shows minimal variation, with less than a 4% change. The results suggest that the developed PVC-based sensing device could be seamlessly integrated into specialized micro-system.

### 3.6. Applications of CHIKV Spiked Serum Sample Analysis

**Figure 6:**
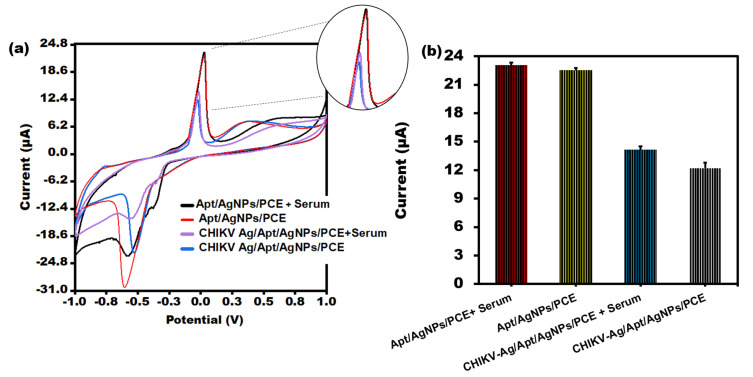
(**a**) CV peak current analysis of CHIKV-Ag in serum sample of humans utilizing PVC sheet-based sensor. (**b**) Bar graph of CHIKV-Ag spiked in serum sample with error bars of standard deviation (*n* = 5).

To assess the clinical applicability of the biosensor under investigation, the maximum change in current was measured in the serum samples containing chikungunya antigen. CV was conducted in a [Fe(CN)_6_]^3−/4−^ solution following the incubation of the Aptamer/AgNPs/PCE sensor in human serum samples containing CHIKV-Ag (10 ng/mL), as illustrated in Figure 6a. The developed sensor successfully identified CHIKV-Ag in human serum, as evidenced by a significant current response comparable to that of the CHIKV-Ag alone. Notably, the current response obtained from human serum alone closely resembled that of the Aptamer/AgNPs/PCE configuration. The aptasensor exhibited sensitivity to samples (serum) and demonstrated a rapid response, as depicted in Figure 6a. Sufficient data were gathered for sensing with actual samples, as illustrated in Figure 6b.

## 4. Comparative Analysis

Several electrochemical biosensors have been developed to detect CHIKV, and the present study has been compared with previously documented techniques for CHIKV detection. Based on their dynamic range and detection limit, Table 3 compares the diagnostic performance of many biosensors for CHIKV. Yupapin et al. [40] developed an Ag-silicon-graphene-based SPR sensor to identify the CHIKV. Sharma and co-workers [41] mentioned using a photonic crystal-based biosensor for chikungunya virus detection with a sensitivity of 3430 nm/RIU. Shukla et al. [42] mentioned developing a multi-layered structure based on aluminum for CHIKV identification at a wavelength of 1550 nm. Using Si/TiN, Daher et al. [43] created a wavelength-based sensor that can detect the chikungunya virus with a sensitivity of up to 2833 nm/RIU. Ag-Silicon-PtSe2-based SPR sensors were reported by Singh et al. [44] to have a maximum sensitivity of 287.3°/RIU for the detection of the CHIKV; however, careful investigation revealed that these sensors needed expensive equipment, powerful energy sources, and sample pre-treatment [45]. Additionally, other investigations attempted to build an immunosensor based on antibodies to detect the CHIKV antigen [46,47]. In our present study, we created a carbon ink-printed PVC electrode-based aptasensor to detect CHIKV. Comparing aptamers to antibodies with similar sensitivities, the former shows more stability [48]. Thus, this integrated sensor intends to layout flexibility, allowing it to bend, have minimum sample requirement, have higher durability and be low-cost (Table 3). Our research has demonstrated analytical effectiveness that either exceeds or matches that of existing biosensors.

## 5. Conclusions and Future Perspectives

This research explained a method for detecting CHIKV-Ag using PVC sheet-based electrochemical biosensors. This article addresses aptamer sequences that can bind to CHIKV. The use of aptamer in conjunction with AgNPs demonstrated diagnostic potential. The biosensor successfully detected CHIKV-Ag in commercial blood samples, showcasing its sensitivity. Under optimal conditions, the proposed PVC sheet-based sensor could detect CHIKV within a linear range of 0.1 ng/mL to 1μg/mL and LOD of 0.1 ng/mL. Moreover, the sensor also showed selectivity against the Dengue virus antigen. These devices can be made inexpensively and are a reliable replacement for traditional ELISA and PCR testing. PVC sheets are an inexpensive and readily available material for sensors. PVC sheets also have high thermal stability, which is essential for sensors operating in extreme temperatures. Additionally, they are resistant to corrosion, which improves their longer shelf life and makes them more reliable when used in electrochemical sensors. Moreover, they are easy to fabricate and have good electrical conductivity. Using a PVC sheet as an electrode in electrochemical sensors has the potential to revolutionize the industry, offering unmatched accuracy and efficiency.

## Figures and Tables

**Table 1:** Recovery test conducted on CHIKV-Ag biosensor.

Initial Concentration µg/mL	Current Measured (µA)	Concentration (Added) µg/mL	Final Current Measured (µA) Mean± SD	Found Concentration µg/mL	RSD (%)	Recovery (%)
0.001	16.00	0.005	14.58 ± 0.27	0.0067	1.91	91
0.01	12.21	0.05	11.30 ± 0.81	0.054	7.1	92.5
0.0001	18.74	0.0005	17.51 ± 0.71	0.0007	4.05	93

**Table 2:** Precision (*n* = 5) assessment of CHIKV-Ag detection using the Aptamer/AgNPs/PCE platform.

Antigen	Within Batch *(n* = 5) Mean ± SD	Between Batch (*n* = 5) Mean ± SD
CHIKV-Ag (ng/mL)	0.15 ± 0.03	0.13 ± 0.01

**Table 3:** The efficiency of different biosensors for detecting CHIKV is compared using the LOD and the dynamic range.

S.No.	Method	Substrate Type	LOD	Linear Range	Reference
1.	Surface plasmon resonance sensor	Silicon and graphene layers are positioned over the base of a glass prism sputtered with a silver coating.	393 degRIU^−1^ (platelets) 160 degRIU^−1^ (plasma cells)	-	[40]
2.	Surface plasmon resonance sensor	BK7prism(coupling)/ Ag(metal)/PtSe_2_(TMD)	287.3 degRIU^−1^	-	[44]
4	Piezoelectric sensing	Cantilever	-	-	[49]
5	Electrochemical biosensor	Three-electrode (conventional)	0.062 pfuml^−1^	-	[50]
6	Immunosensor	Gold (Au) electrode	22.1 fg mL^−1^	100 fg mL^−1^–1 ng/mL^−1^	[46]
7	Genosensor (Electrochemical)	ePAD	0.1 nM	0.1 nM–100 μM	[51]
8	Electrochemical PVC–based sensor	PVC	0.1 ng/mL	0.1 ng/mL to 1 µg/mL	Present Work

## Data Availability

Data will be made available on request.
